# Thermal comfort in relation to housing and tuberculosis status among individuals aged ≥15 years in Padang, West Sumatra Province, Indonesia

**DOI:** 10.3389/fpubh.2026.1868280

**Published:** 2026-06-24

**Authors:** Ringga Rahmi Prima, Alan F. Geater, Virasakdi Chongsuvivatwong, Juntakan Taweekun, Defriman Djafri

**Affiliations:** 1 Department of Epidemiology, Faculty of Medicine, Prince of Songkla University, Songkhla, Thailand; 2 Department of Mechanical and Mechatronics Engineering, Faculty of Engineering, Prince of Songkla University, Songkhla, Thailand; 3 Department of Epidemiology and Biostatistics, Faculty of Public Health, Andalas University, Padang, Indonesia

**Keywords:** Indonesia, indoor environment, thermal perception, thermal sensation vote, tropical, tuberculosis

## Abstract

**Background:**

Tuberculosis (TB) may influence thermal comfort perception through physiological changes such as respiratory symptoms and reduced metabolic activity. This study examined subjective (Thermal Sensation Vote, TSV) and objective (Predictive Mean Vote, PMV) comfort among TB patients, non-TB individuals with respiratory symptoms, and community controls in tropical households.

**Methods:**

This research was designed as a comparative cross-sectional study conducted on a sample initially assembled for a case–control study. We compared TB patients (*n* = 78), non-TB individuals with respiratory symptoms (*n* = 92), and non-respiratory individuals (*n* = 97) recruited from six Puskesmas in Padang, Indonesia (September–December 2024). Inclusion criteria were age ≥15 years having had a previous TB patient in the household but themselves not having been previously diagnosed with TB. Individuals who could not communicate effectively were excluded. Only one participant per household was included. During home visits, environmental parameters (room-air CO₂, humidity, temperature, air speed), body composition, clothing insulation, and thermal comfort indices (TSV, PMV) were assessed. TSV responses were grouped into the broad categories of “comfortable” (slightly cool or neutral) and “too warm” (slightly warm or warm), and associations identified using logistic regression and expressed as odds ratios (and 95% confidence intervals).

**Results:**

The proportion reporting “too warm” exceeded 80% in community and symptomatic groups but was lower among TB patients (69%). Using logistic regression, higher temperature, lower humidity, modern style of house and attached house were all positively associated with feeling too warm. After adjustment for these factors, being a TB patient was associated with a reduced odds of reporting “too warm” [OR = 0.27 (95% confidence interval: 0.10, 0.72)] compared with the community control group.

**Conclusion:**

TB patients reported comfort even under warmer conditions in which most others felt too warm, suggesting altered thermal perception is linked to health status. Indoor climate should be integrated into TB care and housing programs, particularly in tropical settings.

## 1 Introduction

Comfort is recognized as one of the indicators of human wellbeing in built environments (1). A comfortable house not only leads to happiness but also contributes directly to the general health of its occupants. In architecture and environmental health studies, comfort has been examined across several settings, including households, hospitals, schools, and public transportation systems. The perception of comfort influences both psychological satisfaction and physical health outcomes (2–4).

Thermal comfort, in particular, has been defined as the condition in which individuals feel content and satisfied with their thermal environment, characterized by the absence of discomfort ranging from uncomfortably warm to uncomfortably cold. To assess thermal comfort, both subjective and objective measurements are commonly used. Two indicators that are used are the Thermal Sensation Vote (TSV) and the Predicted Mean Vote (PMV) (5–7).

TSV reflects what occupants actually feel at the time of a survey. It captures personal comfort perceptions shaped by culture, health status, psychological state, and adaptation (4, 8, 9). Because it is based on self-reported sensation, TSV is subjective and varies across individuals (2).

In contrast, PMV is an objective index calculated from environmental and personal parameters, including air temperature, relative humidity, air velocity, metabolic rate, and clothing insulation. PMV represents the expected thermal sensation under standardized conditions and is applied in building design and environmental monitoring (10, 11). However, while it may provide an objective scale of thermal comfort, it was developed from data of Danish populations (12) and may not be appropriately calibrated for a tropical setting. Nevertheless, together, TSV and PMV provide complementary insights into thermal comfort, which combine personal feeling and measurable environmental factors (3, 10, 11). Regional and seasonal climatic differences are expected to influence TSV as well as the particular factors related to thermal comfort under different climatic conditions (13), including in Padang, West Sumatra, a tropical area (14), where the current study was conducted.

The weather also influences specific types of housing construction. In Padang, houses have traditionally been designed to prevent dangerous conditions (such as protection from predatory animals in earlier times and protection from earthquakes), to maintain comfort for the occupants, and to address particular situations. Minangkabau culture further shapes housing design, making the form and building materials unique compared to those of other cultures in other countries or even other provinces in Indonesia (15).

In addition to being subject to the effect of different climatic conditions, thermal comfort may also be influenced by an individual’s health status (16). Health conditions can shape whether people feel comfortable or distressed in their surroundings (17). Tuberculosis, caused by infection with *Mycobacterium tuberculosis* (Mtb) (18), is commonly accompanied with low grade fever, as well as poor nutrition and underweight status (19). Both fever (20) and underweight condition (21) have been reported to be conditions leading to imbalance of thermal regulation.

TB treatment has an impact on the overall quality of life (QOL) of the patient (22), which leads to the patient experiencing pain or discomfort (23) and various symptoms that disrupt daily living (24), although these effects usually improve when treatment is completed (25). This discomfort or decreased QOL may be related to the immune response to Mtb infection or to the side effects of TB treatment (26); these physiological changes may be associated with altered thermal perception (27).

Thus, TB patients may perceive comfort differently from healthy individuals (16). Understanding these perceptions is important for management of TB infections as they may influence how a patient experiences and adapts to the living environment. Using TB disease as the focus of interest, the aim of the current study was to compare thermal perceptions including subjective (TSV) and objective (PMV) thermal comfort among TB patients, non-TB individuals with respiratory symptoms, and the general non-respiratory community, and identify housing conditions related to these perceptions, in households in Padang, West Sumatra Province.

## 2 Materials and methods

### 2.1 Study design and population

This research was designed as a comparative cross-sectional study conducted on a sample initially assembled for a case–control study. The aim of the current study was to examine whether the thermal perception of patients diagnosed with tuberculosis differs from that of the general community and patients with non-TB respiratory symptoms. Data were collected from September to December 2024 in Padang City, West Sumatra.

### 2.2 Study area

The study was conducted in Padang City, the capital of West Sumatra Province, Indonesia. Padang has a population of approximately 942,938 and a land area of 694.96 km^2^. The city has a tropical rainforest climate, with average annual temperatures around 26 °C and daily maximum temperatures close to 30 °C. The mean relative humidity is typically high, ranging from 80 to 85%, with little seasonal variation. During the rainy season, RH may rise slightly, but the difference is small compared to the large changes in rainfall totals. The mean monthly rainfall exceeds 400 mm during the rainy season (14). Padang City has 24 primary health care units (Puskesmas), from which six with high TB incidence, namely Andalas, Alai, Kuranji, Pauh, Rawang, and Nanggalo, were selected for this study.

### 2.3 Participant selection and inclusion/exclusion criteria

#### 2.3.1 Inclusion

Individuals attending one or other of the selected Puskesmas and age ≥15 years were screened for living in a household in which another member had previously been a TB patient. Screening was conducted by the admission staff or the research team. Three groups of participants were enrolled; confirmed TB-positive and confirmed TB-negative patients were enrolled from the TB clinics, and general community individuals without any respiratory symptoms were enrolled from general clinics of the Puskesmas. The TB patients included in our study were newly diagnosed cases, defined as individuals found TB-positive using GeneXpert MTB/RIF, and non-TB respiratory symptom patients were those confirmed as TB-negative by GeneXpert MTB/RIF, within the previous month. They were selected after confirming that there had previously been a TB patient in their home and agreeing to join the study. Community controls were enrolled from individuals attending general (non-respiratory) clinics after individually reporting that they had no respiratory symptoms, that they lived in a home where there had previously been a member diagnosed with TB, and that they agreed to join the study. Only one participant from any one household was included.

#### 2.3.2 Exclusion

Participants with a previous history of TB were excluded, not counting the current diagnosis among those selected as cases. Additional exclusions were individuals who could not communicate effectively during the household visit, based on the researcher’s observation and inability to complete the interview. No specific diagnostic measurement for the inability to communicate was used.

### 2.4 Data collection and variables

Participants were recruited directly at the Puskesmas or via telephone by trained research staff in collaboration with TB program officers. After providing written informed consent, all participants were interviewed using a structured questionnaire covering sociodemographic factors, individual vulnerabilities, household characteristics, and previous TB exposure, and a home-visit subsequently made.

The questionnaire was developed specifically for this study, but with the part on thermal comfort modified from the American Society of Heating, Refrigeration and Air-Conditioning Engineers (ASHRAE) Standard 55–2017 (10, 11). Questions on cooking fuel use and household structure items were modified from the Indonesian Health Survey (SDKI) (28).

#### 2.4.1 Individual characteristic variables

At the interview, individual-level variables were collected for all participants. These included sex (female, male), age (15–22 years, 23–59 years, ≥60 years), marital status (single, married), occupation (indoor, outdoor), educational level (primary/junior school, ≥high school), monthly income (<153 USD, 153–306 USD, >306 USD), and religion (Islam, non-Islam). At the same time, an appointment was made for a home visit. At this visit, additional individual characteristics at the actual time of the visit were assessed, and included activity (sleeping/resting/sitting, standing/light activity), clothing insulation (0.3 clo, 0.5 clo, 0.7 clo), and body mass index (BMI) (underweight, normal, overweight/obese). According to the World Health Organization (WHO) standards 2018 for Asian populations, BMI was categorized as underweight (<18.5 kg/m^2^), normal (18.50–24.99 kg/m^2^), overweight (≥25 kg/m^2^), and obese (≥30 kg/m^2^) (29, 30).

#### 2.4.2 Household conditions

Household conditions were measured/recorded at the home visit. These included household structural variables including house design (modern/traditional), type (attached/detached), house stories, floor elevations, construction materials of floor, walls, roof, ceiling, living room windows, living room dimensions, signs of dampness, cooking fuel used, normally having someone smoking in the home/living room, and numbers of persons currently and usually occupying the living room. An attached house was defined as a house that shared at least one side wall with a neighboring house. A detached house was defined as a house that did not share any wall with a neighboring house. House type was also categorized as modern (cement-based) or traditional (constructed mainly of wood and in a local semi-traditional style).

The following environmental parameters in the living room of the house, and the instruments used, were measured.

1. *Temperature and relative humidity:* Peak Meter PM6508 Digital Temperature and Humidity Meter (temperature range: −20 to 60 °C, precision: ±0.5 °C; humidity range: 0–100% RH, accuracy: ±2.0% RH).
2. *Wind speed:* Handheld Anemometer Digital Wind Speed Meter (GM816 Pocket LCD Digital Anemometer) (range: 0–30 m/s, accuracy: ±5%). Measurements were performed at each visit using a portable mini-fan (Model: RT-BF25) to generate a low-speed reference airflow. Natural air speed was then measured and interpreted relative to this reference.
3. *Carbon dioxide (CO₂) concentration* was measured as an indicator of ventilation using Sndway SW723 Carbon Dioxide Detector (range: 0–9,999 ppm, resolution: 1 ppm).

All instruments were held at chest level (approximately 1.2–1.3 m). Windows and doors were left in their current state. Each reading was recorded after the digital display had stabilized (3–5 s). Measurement times were scheduled according to participant availability, typically during daytime hours (08:00–17:00), in the living room of each participant’s house. This one-time measurement was used to relate to the thermal sensation at the exact same time.

#### 2.4.3 Thermal comfort assessment

Thermal comfort measures included TSV and PMV. During home visits, participants reported their immediate feelings by recording a TSV on the seven-point ASHRAE scale (cold, cool, slightly cool, neutral, slightly warm, warm, hot) (10, 11).

PMV was calculated using the “pythermalcomfort” Python package, which complies with and adheres to international standards (ASHRAE 55–2017, ISO 7730:2005, European Norm {EN 16798–1:2019}) (31) and is consistent with the Centre for the Built Environment (CBE) Thermal Comfort Tool (32). PMV estimation incorporates both environmental variables (air temperature, air speed, relative humidity) and personal variables (metabolic rate (MET) and clothing insulation). Metabolic rate was estimated from the activity level (sitting, walking or sleeping) and clothing insulation based on the type of clothing they were wearing during the home visit (10, 11).

Thus, TSV reflects participants’ subjective thermal sensations, while PMV represents the model’s prediction based on measurable physical conditions (11).

### 2.5 Sample size consideration

Since this study was based on the sample used in a previous case–control protocol, the detectable effect size proportion of feeling comfortable neutral in each group was calculated using a two-sample comparison of independent proportions between the general community and TB groups. It was unknown if the TB group would have a lower or higher proportion of participants feeling comfortable. With the sample size available (97, 92, and 78 in community, symptomatic and TB groups respectively), and an assumed proportion of feeling comfortable among the general community of 15%, significant disparities could be detected if the proportion in the TB group were to be ≤2% or ≥34% at alpha = 0.05 and beta = 0.2. Corresponding TB values for an assumed proportion in the community of 20% would be ≤5% or ≥41%.

### 2.6 Data management and analysis

The questionnaire was developed in English, translated into the Indonesian language and the interview conducted in the Indonesian language. All data were recorded using Kobo Toolbox (33). The final review for data cleaning, missing data, and error checking was done using STATA release 18 (34).

Categorical variables were reported as frequencies and percentages, while continuous variables were reported as means with standard deviations. Differences between the general community, non-TB symptomatic and TB-positive patient groups were assessed using chi-square tests for categorical variables and either *t*-tests or Wilcoxon rank-sum tests for continuous variables.

Owing to the small numbers of patients in some TSV categories, the levels were collapsed into a binary variable comprising comfortable and too warm, and individual and household characteristics and home visit measurements compared across these thermal perception groups.

To visualize environmental conditions, kernel density estimation (KDE) was applied to describe the joint distribution of air temperature (Ta) and relative humidity (RH). KDE contour plots were generated in Python.

A multivariable logistic regression model identifying health status, individual, room air, and household variables was compiled sequentially to estimate associations with thermal comfort. In Stage 1, health status was included alone. In Stage 2, individual variables (sex, marital status, and monthly income) were added. In Stage 3, room air parameters (temperature, relative humidity, CO₂) were incorporated, while month of visit was excluded due to collinearity with temperature. In Stage 4, house type (modern vs. traditional, attached vs. detached) was added, with structural materials excluded owing to collinearity with house type. Variables were retained if their removal increased the AIC, and associations were expressed as odds ratios (OR) with 95% confidence intervals.

### 2.7 Ethical approval

This study adhered to the principles of the Helsinki Declaration. Ethical approval for this study was obtained from the Ethics Committee of the Padang City Government Office (EC Number: 070.11749/DPMPTSPPP/VIII/2024). The study protocol was submitted through the authority’s online system, reviewed by the committee, and approval notification was issued via email to the researcher. All participants provided informed consent prior to inclusion in the study.

## 3 Results

### 3.1 Descriptive statistics

A total of 267 participants were enrolled, including 78 TB patients, 92 non-TB individuals with respiratory symptoms, and 97 from the general community. Individual and house characteristics of each of the 3 groups are shown in Supplementary Tables S1, S2. Across all groups, females, age 23 years and older, indoor occupation, Islamic religion and a normal BMI predominated. Among housing characteristics, attached housing, modern style, with cement walls, metal roofs and wooden ceilings, were most frequent. At the time of the home visit, most participants were sitting or resting. Measured living room air temperature ranged from 25.6 to 35.0 °C (mean 30.9 °C), and measured relative humidity ranged from 60 to 93% (mean 71.6%).

Table 1 shows the distribution of subjective thermal sensation (TSV) among the study groups. In all groups, the most commonly reported feeling was slightly warm (69.1, 73.9, and 50.0% in the general community, symptomatic and TB-positive groups, respectively). Feeling slightly warm or too warm comprised over 80% in the general community and symptomatic groups, but a significantly lower proportion (69.2%) of the TB-positive participants. Of all groups, the TB group reported the highest proportion of feeling neutral (26.9%). The distribution of the collapsed binary thermal perception variable by health status is shown in Table 2.

**Table 1:** TSV according to health status.

Health status	Slightly cool (*n* = 10)	Neutral (*n* = 40)	Slightly warm (*n* = 174)	Warm (*n* = 43)	*p*-value^*^
TB status					<0.001
General Community	4 (4.1)	8 (8.3)	67 (69.1)	18 (18.6)	
Non-TB symptomatic	3 (3.3)	11 (12.0)	68 (73.9)	10 (10.9)	
TB positive	3 (3.9)	21 (26.9)	39 (50.0)	15 (19.2)	

*Chi-square test.

**Table 2:** Thermal perception according to health status.

Health status	Comfortable (*n* = 50)	Too warm (*n* = 217)	*p*-value*
TB status			<0.001
General community ^a^	12 (12.4)	85 (87.6)	
Non-TB symptomatic ^a^	14 (15.2)	78 (84.8)	
TB positive ^b^	24 (30.8)	54 (69.2)	

Groups not having a superscript in common differ significantly at the 5% level (Wald test). *Chi-square test.

#### 3.1.1 Individual static variables and variables pertaining to the time of the home visit

Table 3 compares individual characteristics between participants who reported feeling comfortable and those who felt “too warm.” Apart from TB status, the only near significant difference was found for the sex of the participant. Females were more likely than males to report feeling too warm (83.8% vs. 73.9%, *p* = 0.069).

**Table 3:** Individual characteristics of participants according to thermal perception (number and row percentage).

Variable	Comfortable (*n* = 50)	Too warm (*n* = 217)	*P*-value*
A. Individual static variables			
Sex			0.069
Female	32 (16.2)	166 (83.8)	
Male	18 (26.1)	51 (73.9)	
Age			
15–22 years	5 (21.7)	18 (78.3)	
23–59 years	38 (20.7)	146 (79.3)	
60 + years	7 (11.7)	53 (88.3)	
Marital status			0.076
Single	14 (27.5)	37 (72.6)	
Married	36 (16.7)	180 (83.3)	
Occupation			0.179
Indoor	44 (17.8)	203 (82.2)	
Outdoor	6 (30.0)	14 (70.0)	
Educational level			0.402
Primary and junior school	12 (15.6)	65 (84.4)	
≥High school	38 (20.0)	152 (80.0)	
Monthly income (USD)			0.166
<153	38 (21.2)	141 (78.8)	
153–306	8 (11.3)	63 (88.7)	
>306	4 (23.5)	13 (76.5)	
Religion			0.785
Non-Islam	2 (22.2)	7 (77.8)	
Islam	48 (18.6)	210 (81.4)	
B. Variables pertaining to the time of the home visit			
Body composition			
Metabolic rate (MET)*			0.733
Sleeping/resting/sitting (1.0 MET)	44 (19.1)	187 (80.9)	
Standing/light activity (1.5 MET)	6 (16.7)	30 (83.3)	
BMI			0.387
Underweight	9 (23.1)	30 (76.9)	
Normal	29 (20.3)	114 (79.7)	
Overweight/obese	12 (14.1)	73 (85.9)	
Clothing insulation (n)			0.580
Trousers, short-sleeve shirt (0.3 clo)	20 (17.2)	96 (82.8)	
Trousers, long-sleeve/dress Muslim clothes (0.5 clo)	30 (20.3)	118 (79.7)	
Trousers, knit, jacket (0.7 clo)	0 (0.0)	3 (100.0)	

*Chi-squared *p*-value. Outdoor occupation (agriculture/fishery, labor/manual work). Indoor occupation (student/retired/homemaker/housewife/civil servant/state enterprise). Metabolic rate (met): 1 met = 58.2 W/m^2^ (energy produced per unit body surface area by a seated person at rest). Sitting ≈ 1.0 met, standing/light activity ≈ 1.5 met. Clothing insulation (clo): 1 clo = 0.155 m^2^·K/W. Categories: short-sleeve ≈ 0.5 clo, long-sleeve/Muslim dress ≈ 0.7–0.9 clo, jacket ≈ 1.0 clo.

#### 3.1.2 Housing structural characteristics

Several housing characteristics differed significantly between thermal perception groups (Table 4). Participants living in a modern-style house, or in an attached house, more commonly reported feeling too warm compared to those living in traditional or in detached houses, respectively, (85.6% vs. 59.1 and 83.1% vs. 46.2%, each *p* < 0.001). Other housing features, such as the number of stories, floor elevation, orientation, and cooking fuel, showed no significant differences.

**Table 4:** Housing characteristics according to participants’ thermal perception (number and row percentage).

Variable	Comfortable (*n* = 50)	Too warm (*n* = 217)	*P*-value*
A. Household structural variables			
House type			<0.001
Traditional	18 (40.9)	26 (59.1)	
Modern	32 (14.4)	191 (85.6)	
House separated/attached			<0.001
Attached	43 (16.9)	211 (83.1)	
Detached	7 (53.9)	6 (46.2)	
House stories			0.523
1 Story	47 (19.2)	198 (80.8)	
2 Stories	3 (13.6)	19 (86.4)	
Floor elevation			0.514
On ground	46 (19.9)	185 (80.1)	
1 Meter	3 (13.6)	19 (86.4)	
2 Meters	1 (14.3)	6 (85.7)	
3 Meters	0 (0.0)	7 (100.0)	
House orientation (front-facing direction)			0.149
North	7 (19.4)	29 (80.6)	
Northeast	2 (8.0)	23 (92.0)	
East	8 (19.5)	33 (80.5)	
Southeast	5 (17.2)	24 (82.8)	
South	7 (17.1)	34 (82.9)	
Southwest	9 (30.0)	21 (70.0)	
West	4 (9.5)	38 (90.5)	
Northwest	8 (34.8)	15 (65.2)	
Cooking fuel			0.751
Clean fuel	48 (18.9)	206 (81.1)	
Dirty fuel	2 (15.4)	11 (84.6)	
Roof material			0.254
Tile/asphalt/+	1 (7.1)	13 (92.9)	
Metal	49 (19.4)	204 (80.6)	
Ceiling material			0.069
Gypsum/plaster	8 (32.0)	17 (68.0)	
Wood	33 (15.9)	175 (84.1)	
Other	9 (26.5)	25 (73.5)	
Floor material			0.997
Cement	17 (18.5)	75 (81.5)	
Wood/other	3 (18.8)	13 (81.3)	
Ceramic	30 (18.9)	129 (81.1)	
Wall material			0.144
Cement	33 (16.2)	171 (83.8)	
Wood	15 (26.3)	42 (73.7)	
Ceramic/other	2 (33.3)	4 (66.7)	
Living room volume (m^3^)			0.599
≥9	36 (18.0)	164 (82.0)	
<9	14 (20.9)	53 (79.1)	
Living room windows opened			0.768
Yes	43 (19.0)	183 (81.0)	
Rarely/none	7 (17.1)	34 (82.9)	
B. Variables related to home visit			
Month			<0.001
September	9 (26.5)	25 (73.5)	
October	9 (9.5)	86 (90.5)	
November	7 (8.8)	73 (91.3)	
December	25 (43.1)	33 (56.9)	
Relative humidity (%)			<0.001
(Mean ± SD)	76.9 ± 6.9	70.3 ± 5.3	
Air temperature (°C)			<0.001
(Mean ± SD)	28.4 ± 1.8	31.5 ± 2.0	
Air speed (m/s)			0.549
(Mean ± SD)	0.07 ± 0.30	0.11 ± 0.36	
CO₂ level (ppm)			0.060
(Mean ± SD)	572.9 ± 92.9	546.0 ± 89.7	
Predictive mean vote (PMV) (objective thermal comfort)			<0.001
(Mean ± SD)	1.00 ± 0.85	2.14 ± 0.86	

*Chi-squared *p*-value. Clean fuel (electricity, gas). Dirty fuel (wood, kerosene). Tile/asphalt + (tile, asphalt or other non-metal). Fanger’s Predicted Mean Vote (PMV): Dimensionless index (−3 cold to +3 hot) derived from air temperature, air speed, relative humidity, metabolic rate (met), and clothing insulation (clo). PMV=(0.303×exp(−0.036M)+0.028)×L.

#### 3.1.3 Home-visit variables

Means of measured living room air temperature differed significantly across the months of the home visit (*p* < 0.001). Over 90% of participants reported feeling too warm when home visits were made in October or November, with mean reported temperatures of 31.9 and 31.0 °C respectively, followed by September (74%) and December (57%) with mean reported temperatures of 30.2 and 29.5 °C, respectively. Measured room air temperature was higher (31.5 °C vs. 28.4 °C) and relative humidity lower (70.3% vs. 77.1%) in houses of participants who reported feeling too warm (Table 4).

Among other home-visit variables, only CO_2_ showed some evidence of a difference between the thermal perception groups, higher in the comfortable than in the too warm households. PMV, which incorporates various environmental variables, was significantly higher among the too-warm participants than among those who felt comfortable (2.14 vs. 1.00 *p* < 0.001).

**Figure 1:**
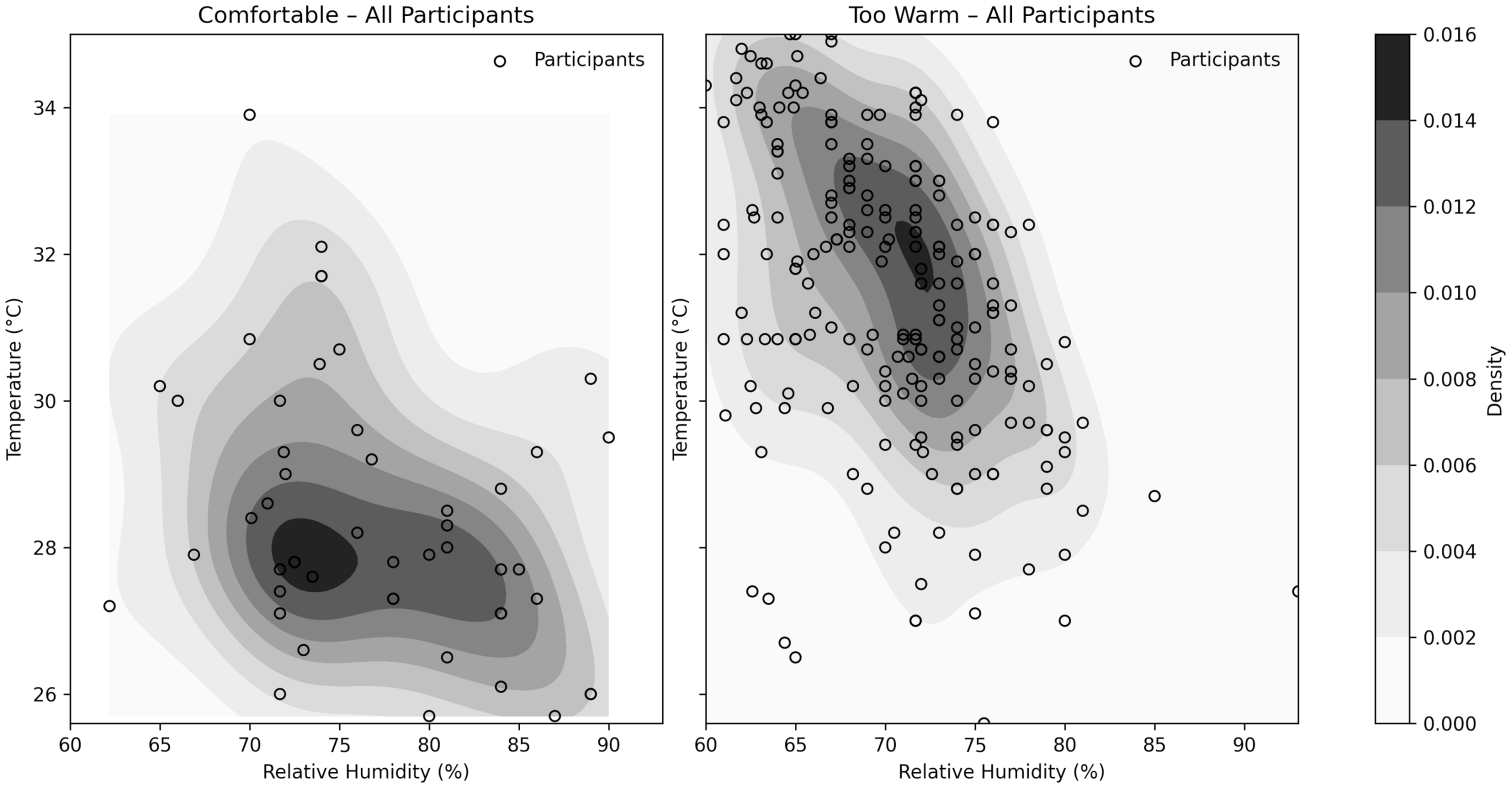
Kernel density contour plots of temperature and relative humidity by thermal perception.

**Figure 2:**
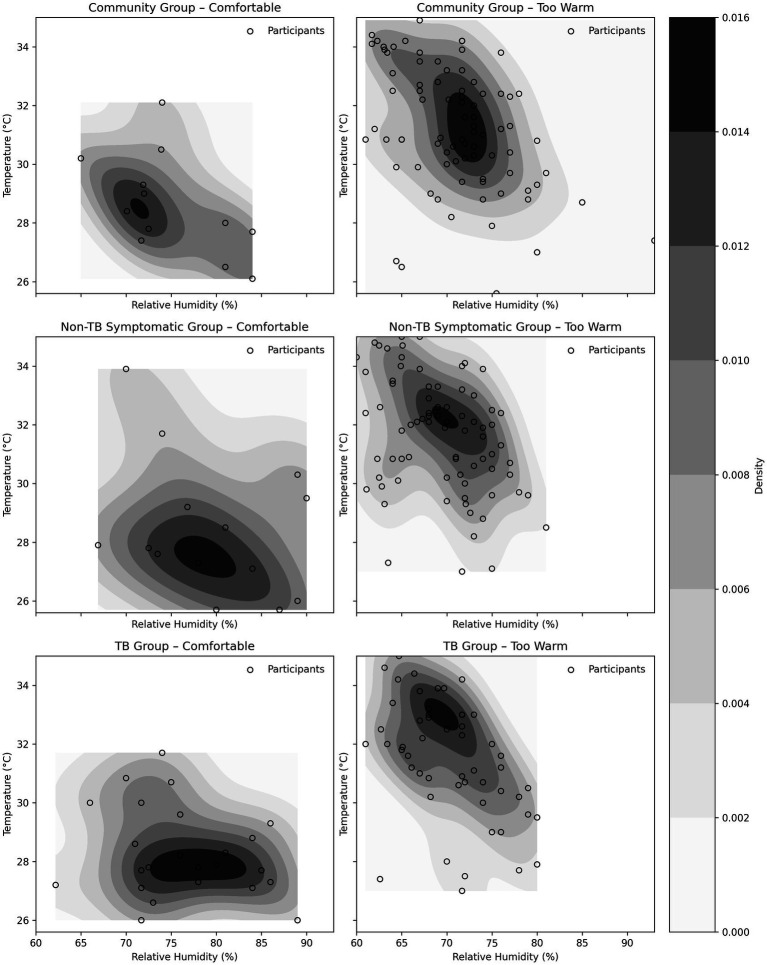
Kernel density contour plots of temperature and relative humidity by group and thermal perception.

The combined distributions of room air temperature and relative humidity in the comfortable and too-warm perception groups are depicted in Figure 1 in which contours of point spatial kernel density are displayed. Overall, participants feeling too warm were mostly found in houses in which the room air temperatures were higher and relative humidity within a lower and narrower range. Figure 2 shows that among participants feeling too warm the peak of the temperature density distribution was higher in the TB group than in the community group, while among the comfortable participants the peak of the RH distribution was higher in the TB and non-TB symptomatic groups than that in the community group. Linear regression models including an interaction term between health group and thermal perception group supported these differences in temperature, relative humidity, as well as PMV, across health and thermal perception subgroups (Supplementary Table S3).

### 3.2 Multivariable analysis

The logistic regression model is shown in Table 5. None of the individual variables was significant when added to the model containing health group. However, the addition of temperature and humidity as potential confounding variables significantly improved the fit of the model, as did the addition of house type variables, namely modern vs. traditional and attached vs. detached. At each stage of the model assembly, the TB positive group was significantly less likely to feel too warm compared to the general community group [OR = 0.27 (0.10, 0.72) in the full model], although not significantly so compared with the non-TB symptomatic group.

**Table 5:** Effect of TB status and associated factors on “Too Warm” versus comfortable response.

Factor	Category	Stage 1		Stage 2		Stage 3	
		OR (95% CI)	LR test *p-*value	OR (95% CI)	LR test *p-*value	OR (95% CI)	LR test *p-*value
Group	General community	1 ^a^	0.007	1^a^	0.030	1 ^a^	0.027
	Non-TB symptomatic	0.79 (0.34, 1.80) ^a^		0.63 (0.23, 1.72) ^a^		0.51 (0.17, 1.50) ^ab^	
	TB positive	0.32 (0.15, 0.69) ^b^		0.29 (0.11, 0.75) ^b^		0.27 (0.10, 0.72) ^b^	
Environment	Temperature, °C			1.89 (1.51, 2.37)	<0.001	1.78 (1.41, 2.25)	<0.001
	Relative humidity, %			0.91 (0.85, 0.97)		0.89 (0.82, 0.95)	
House type	Traditional wooden					1	0.006
	Modern					2.40 (0.92, 6.21)	
	Detached					1	
	Attached					6.22 (1.21, 31.85)	
AIC		253.36		173.30		166.94	

Independent covariables. Stage 1: Health status only. None of the individual variables added at this stage was statistically significant or reduced the AIC. Stage 2: Addition of relative humidity (%) + air temperature (°C). Stage 3: Addition of house type (modern/traditional house + separate/attached). Groups not having a superscript in common within group in each model differ significantly at the 5% level (Wald test).

Higher temperature [OR per ^o^C increase = 1.78 (1.41, 2.25)], lower relative humidity [OR per % increase = 0.89 (0.82, 0.95)] (LR *p* < 0.001), modern style of house [OR = 2.40 (0.92, 6.21)] and attached house [OR = 6.22 (1.21, 31.85)] were positively associated with feeling too warm (LR *p* = 0.006).

## 4 Discussion

This study has revealed a difference in thermal perception between TB patients and the general community in this tropical TB-endemic setting. The concept of thermal comfort was originally developed based on the Danish population. The TSV scale is purely subjective, while the more objective PMV scale may not be directly applicable to climatic settings, such as our tropical setting, that differ from that of Denmark (12). The previous study was conducted as a laboratory experiment with individuals living in a 4-season controlled climate; however, our study was based on home visits where thermal comfort was assessed under natural household conditions.

Overall, around 80% of the participants reported feeling “too warm” and this can be explained by the fact that Padang City is located near the equator and has a tropical climate (14) and the observation that most people in this area tend to open windows to feel comfortable and rarely use air conditioning (13).

Females in Muslim culture tend to wear more fully covered clothing (35) and this is consistent with the somewhat higher percentage of females feeling too warm. However, sex was not significantly associated with thermal comfort in the multivariable model. Regarding religion, 97.8% of Padang residents were Muslim in 2024 (36), which is consistent with the reported religion in our study (96.6% Muslim). Feeling too warm was also more common among participants residing in a modern style house and in an attached house, likely owing to the poorer ventilation offered by those houses.

Of particular interest is the finding that, compared to the other two groups, individuals in the TB group were significantly less likely to feel too warm. This association remained significant even after adjusting for environmental and housing factors, suggesting that TB status itself contributes to differences in comfort perception. The effect could be due to the presence of low grade fever, which is typical of pulmonary TB patients during the early phase of treatment (37). Patients experiencing low grade fever, when the thermal set point is still elevated with respect to body temperature, may feel chilly and exhibit physiological and behavioral responses, such as shivering, putting on extra clothing or seeking warmer environmental conditions (20).

Independently of the health status and of temperature and humidity, feeling too warm was also associated with residence in modern and attached houses. In both of these settings, ventilation is likely to be more limited than in detached and traditional houses, respectively. A similar situation was reported in Danang, Vietnam (38), in which an attached house was described as having limited cross ventilation owing to the side walls being shared with neighbors and therefore having few or no windows. Adequate airflow is essential in residential buildings, reducing heat accumulation in the house structure (39) and enhancing the evaporative cooling of the skin surface of occupants (40); both of these effects play a role in thermal comfort in warm environments.

Modern houses typically have smaller rooms and cement walls that may act as a heat store, unlike the almost entirely wooden structure of a traditional house. Cement walls have slow heat transfer and trap heat inside the building, resulting in higher indoor temperatures compared with timber walls (41). The structural form of traditional houses in Indonesia has developed over a long time to adapt to the hot climate and typically has large openings, long overhangs and uses local materials, mostly wood, for optimizing ventilation and indoor thermal quality. On the other hand, recently, due to increasing populations and demand for housing, the modern house has become a more affordable choice (42). This is also reflected in our sample, in which modern houses were predominant.

While the association between TB status and thermal comfort has been demonstrated, it should be noted that there is some evidence that thermal discomfort may, itself, predispose to certain infections. In this regard, a number of studies have indicated how thermal stress could lead to increased vulnerability to respiratory infection, such as through a decrease in lysozyme concentration in nasal lavage fluid (43) and risk of a dry cough (44). How this may impact the findings of the current cross-sectional study remains unclear. Nevertheless, the difference in how TB patients and non-TB individuals perceive their thermal environment suggests that this might be a factor to be considered in interventions designed to provide optimal conditions for a newly diagnosed TB patient.

Several limitations of this study should be noted. First, the study included only participants who resided in households in which there had been a previous TB patient, which may limit the generalizability of our findings. This should be considered when interpreting our results; such households may pose a higher risk of other members developing active TB, as household members of TB patients often share common risk factors with the patient, including poverty, poor housing, and environmental exposures (45, 46).

Second, the preponderance of females in each health group, but particularly in the non-TB symptomatic and general community groups may not accurately represent the corresponding sex ratios in the population (47). However, in the multivariable model sex was not found to be a significant predictor of thermal sensation.

Third, environmental variables were measured cross-sectionally and do not correspond to the timeline of TB disease development, and the parameters were not measured at the same time of day in each household. As a result, the relationship between thermal comfort and TB captures only a snapshot of household conditions and participants’ perceptions at the time of the home visit. However, the relationship reflects, as intended, the immediate thermal sensation at the exact time that the environmental parameters were recorded, following ASHRAE 55–2017 guideline, rather than an averaged perception over time during which room-air conditions likely fluctuated.

## 5 Conclusion

TB-positive participants were consistently less likely to report feeling “too warm” at temperature at which a majority of the general community felt too warm. Overall, residence in traditional and detached housing was associated with greater thermal comfort.

### 5.1 Policy and practice implications

Policies should recognize thermal comfort as a component of health. Housing programs must address both medical treatment and living conditions to reduce patient stress and improve wellbeing. At the community level, effective interventions require multidisciplinary cooperation among the Urban Planning Department, the TB Program within the Ministry of Health, and local Puskesmas. Together, these stakeholders can promote healthier and more comfortable living environments for TB patients.

## Data Availability

The raw data supporting the conclusions of this article will be made available by the authors, without undue reservation.

## References

[1] Hernandez-Martin M Del Ama GF González-Lezcano RA. Indoor environmental quality to ensure the health and wellbeing of vulnerable people in residential buildings: a systematic review. Front Built Environ. (2025) 11:11. doi: 10.3389/fbuil.2025.1652527

[2] Lala B Biju A Vanshita RA Dahiya K Kala SM Hagishima A. The challenge of multiple thermal comfort prediction models: is TSV enough? Buildings. (2023) 13:15–27. doi: 10.3390/buildings13040890

[3] Pao SL Wu SY Liang JM Huang IJ Guo LY Wu WL . A physiological-signal-based thermal sensation model for indoor environment thermal comfort evaluation. Int J Environ Res Public Health. (2022) 19:7292. doi: 10.3390/ijerph19127292, 35742537 PMC9223375

[4] Arsad FS Hod R Ahmad N Baharom M Ja’afar MH. Assessment of indoor thermal comfort temperature and related behavioural adaptations: a systematic review. Environ Sci Pollut Res. (2023) 30:73137–49. doi: 10.1007/s11356-023-27089-9, 37211568 PMC10287772

[5] Çağlak S Matzarakis A. Evaluation of the relationship between thermal comfort conditions and respiratory diseases in Amasya City. Turkey J Public Health. (2023) 31:2011–20. doi: 10.1007/s10389-023-01887-4, 37361296 PMC10029800

[6] Kumar TMS Kurian CP. Real-time data based thermal comfort prediction leading to temperature setpoint control. J Ambient Intell Human Comput. (2023) 14:12049–60. doi: 10.1007/s12652-022-03754-8

[7] McDowall R. "Thermal comfort". In: Fundamentals of HVAC IP Book. New York: Elsevier (2006). p. 32–42.

[8] Vellei M Chinazzo G Zitting KM Hubbard J. Human thermal perception and time of day: a review. Temperature. (2021) 8:320–41. doi: 10.1080/23328940.2021.1976004, 34901316 PMC8654484

[9] Kang M Kim KR Lee JY Shin JY. Determination of thermal sensation levels for Koreans based on perceived temperature and climate chamber experiments with hot and humid settings. Int J Biometeorol. (2022) 66:1095–107. doi: 10.1007/s00484-022-02261-x, 35244763 PMC9132799

[10] ANSI/ASHRAE. Addendum d to ANSI/ASHRAE standard 55-2017. London: Routledge (2025).

[11] American Society of Heating, Refrigerating and Air-Conditioning Engineers. ASHRAE Handbook: Fundamentals. Atlanta: ASHRAE (2009). p. 2009.

[12] Roelofsen P Jansen K Vink P. A larger statistical basis and a wider application area of the PMV equation in the Fanger model. Intell Build Int. (2022) 14:517–24. doi: 10.1080/17508975.2021.1928595

[13] Damiati SA Zaki SA Rijal HB Wonorahardjo S. Field study on adaptive thermal comfort in office buildings in Malaysia, Indonesia, Singapore, and Japan during hot and humid season. Build Environ. (2016) 109:208–23. doi: 10.1016/j.buildenv.2016.09.024

[14] Padang Statistical Office. Statistical Report Padang 2024. Padang: Padang Statistical Office (2024).

[15] Alimin NN Triatmodjo S. The amalgamation style of Rumah Gadang in architecture and interior of Istano Basa Pagaruyung in Batusangkar, Indonesia: iconography-iconology analysis. Cogent Arts Humanit. (2025) 12:2442165. doi: 10.1080/23311983.2024.2442165

[16] Ormandy D Ezratty V. Health and thermal comfort: from WHO guidance to housing strategies. Energy Policy. (2012) 49:116–21. doi: 10.1016/j.enpol.2011.09.003

[17] Frontczak M Wargocki P. Literature survey on how different factors influence human comfort in indoor environments. Build Environ. (2011) 46:922–37. doi: 10.1016/j.buildenv.2010.10.021

[18] World Health Organization. Global Tuberculosis Report 2025. Geneva: WHO; (2025). Available online at: https://iris.who.int/server/api/core/bitstreams/e97dd6f4-b567-4396-8680-717bac6869a9/content (Accessed February 3, 2026).

[19] Feleke BE Feleke TE Biadglegne F. Nutritional status of tuberculosis patients: a comparative cross-sectional study. BMC Pulm Med. (2019) 19:182. doi: 10.1186/s12890-019-0953-0, 31638950 PMC6802320

[20] Cimpello LB Goldman DL Khine H. Fever pathophysiology. Clin Pediatr. Emerg Med. (2000) 1:84–93. doi: 10.1016/s1522-8401(00)90012-0

[21] He M Guo J Liu H Xiong F Zhou S Wu Y . Does BMI affect thermal comfort in young people in warm indoor environments? Evidence from a climate chamber experiment in non-normal BMIs. J Build Eng. (2025) 106:112618. doi: 10.1016/j.jobe.2025.112618

[22] Aggarwal AN. Quality of life with tuberculosis. J Clin Tuberc Other Mycobact Dis. (2019) 17:100121. doi: 10.1016/j.jctube.2019.100121, 31788563 PMC6880022

[23] Arifin B Sarwadan MG Wahyudin E Sarifah LM Fuady A Purba FD . Stigma and health-related quality of life among people with multidrug-resistant tuberculosis: a cross-sectional study in Indonesia. Narra J. (2025) 5:e1317. doi: 10.52225/narra.v5i2.1317 40951506 PMC12425512

[24] Hansel NN Wu AW Chang B Diette GB. Quality of life in tuberculosis: patient and provider perspectives. Qual Life Res. (2004) 13:639–52. doi: 10.1023/B:QURE.0000021317.12945.f0, 15130027

[25] Febi AR Manu MK Mohapatra AK Praharaj SK Guddattu V. Psychological stress and health-related quality of life among tuberculosis patients: a prospective cohort study. ERJ Open Res. (2021) 7:00251–2021. doi: 10.1183/23120541.00251-2021, 34476253 PMC8405877

[26] Xu Y Wu J Liao S Sun Z. Treating tuberculosis with high doses of anti-TB drugs: mechanisms and outcomes. Ann Clin Microbiol Antimicrob. (2017) 16:67. doi: 10.1186/s12941-017-0239-4, 28974222 PMC5627446

[27] Schieber AM Ayres JS. Thermoregulation as a disease tolerance defense strategy. Pathog Dis. (2016) 74:ftw106. doi: 10.1093/femspd/ftw106, 27815313 PMC5975229

[28] The DHS Program. Indonesia: Standard DHS (2024). Available online at: https://dhsprogram.com/methodology/survey/survey-display-605.cfm (Accessed March 3, 2025).

[29] World Health Organization. European Health Information at Your Fingertips. (2024). Available online at: https://gateway.euro.who.int/en/indicators/mn_survey_19-cut-off-for-bmi-according-to-who-standards/ (Accessed January 3, 2025).

[30] Lu P Zhang Y Liu Q Ding X Kong W Zhu L . Association of BMI, diabetes, and risk of tuberculosis: a population-based prospective cohort. Int J Infect Dis. (2021) 109:168–73. doi: 10.1016/j.ijid.2021.06.053, 34217872

[31] Tartarini F Schiavon S. Pythermalcomfort: a Python package for thermal comfort research. SoftwareX. (2020) 12:100578. doi: 10.1016/j.softx.2020.100578

[32] Tartarini F Schiavon S Cheung T Hoyt T. CBE thermal comfort tool: online tool for thermal comfort calculations and visualizations. SoftwareX. (2020) 12:100563. doi: 10.1016/j.softx.2020.100563

[33] Lakshminarasimhappa MC. Web-Based and Smart Mobile App for Data Collection: Kobo Toolbox/Kobo Collect, vol. 57. Singapore: Springer (2021).

[34] StataCorp. Stata Statistical Software: Release 18. College Station, TX: StataCorp LLC (2023).

[35] Yusof INM Ahmad MR Yusof NA Yahya MF Hussain IA Raja Azidin RMF . Thermal comfort perception of hijab usage among young Muslim women for sports activity. Res J Text Appar. (2020) 25:105–17. doi: 10.1108/RJTA-05-2020-0049

[36] Statistics Indonesia, Padang Municipality. (2026). Population by Religion – Statistical Data. Available online at: https://padangkota.bps.go.id/en/statistics-table/2/MjQxIzI=/jumlah-penduduk-menurut-agama.html (Accessed June 3, 2026).

[37] Yassin Z Ahmadinejad Z. The fever response after treatment of tuberculosis: what is the expected time and what are the risk factors? J Infect Dis Treat. (2016) 2:1–4. doi: 10.21767/2472-1093.100011

[38] Le D Tanaka I Zhang Q Mayama R. Thermal comfort in mixed-mode cooled houses: a field study in the hot-humid climate of Danang. Vietnam Energy Build. (2025) 345:116091. doi: 10.1016/j.enbuild.2025.116091

[39] Suszanowicz D. Optimisation of heat loss through ventilation for residential buildings. Atmos. (2018) 9:95. doi: 10.3390/atmos9030095

[40] Ashworth E. Sweat evaporation in humans: a molecular and thermodynamic perspective. Exp Physiol. (2025) 111:643–52. doi: 10.1113/EP093011, 40719527 PMC12949171

[41] Alegbe M. Comparative analysis of wall materials toward improved thermal comfort, reduced emission, and construction cost in tropical buildings. In: Proceedings of the 11th Masters Conference: People and Buildings. London: University of Westminster; (2022).

[42] Tungka A Syafriny R Sangkertadi T. "Thermal comfort comparison of traditional architecture and modern style housing in North Sulawesi, Indonesia". In: Proceedings of the International Seminar SENVAR 9th & ISESEE 2nd. Malaysia: Kuala Lumpur (2008)

[43] Cheng X Zhou Z Yang C Zheng X Liu C Huang W . The impact of draught on thermal comfort and respiratory immunity. SSRN Electron J. (2022). doi: 10.2139/ssrn.4035519

[44] Sun C Cai G Liu W Zou Z Huang C. Thermal comfort in residences related to respiratory diseases among preschool children in Shanghai. Energ Buildings. (2021) 236:110729. doi: 10.1016/j.enbuild.2021.110729

[45] MacPherson P Lebina L Motsomi K Bosch Z Milovanovic M Ratsela A . Prevalence and risk factors for latent tuberculosis infection among household contacts of index cases in two South African provinces: analysis of baseline data from a cluster-randomised trial. PLoS One. (2020) 15:e0230376. doi: 10.1371/journal.pone.0230376, 32182274 PMC7077873

[46] World Health Organization. (2026). Natural Ventilation for Infection Control in Health Care Settings. Available online at: https://www.who.int/publications/i/item/9789241547857 (Accessed June 3, 2026). 23762969

[47] Statistics Indonesia (BPS). (2026). Percentage of Population Seeking Outpatient Care in the Past Month by Province and Type of Treatment Facility, 2009–2022. Available online at: https://www.bps.go.id/en/statistics-table/1/MTYyMCMx/persentase-penduduk-yang-berobat-jalan-sebulan-terakhir-menurut-provinsi--dan-tempat-cara-berobat--2009-2022.html (Accessed June 3, 2026).

