# Supplementary material for: Thermal comfort in relation to housing and tuberculosis status among individuals aged ≥15 years in Padang, West Sumatra Province, Indonesia
Source: Front Public Health. 2026 Jun 24;14:1868280. doi: 10.3389/fpubh.2026.1868280 (PMC13341609; doi:10.3389/fpubh.2026.1868280)
Supplement: Supplementary file 1 [file Table_1.docx]

**Supplementary Material.**

**Table S1.** Individual characteristics by participant group

| **Variable** | **Community (n=97)** | **Non-TB symptomatic (n=92)** | **TB**  **(n=78)** | **P-value^a^** |
| --- | --- | --- | --- | --- |
| **A, Individual static variables** | |  |  |  |
| **Sex** |  |  |  | <.001 |
| Female | 79 (81.4) | 77 (83.7) | 42 (53.8) |  |
| Male | 18 (18.6) | 15 (16.3) | 36 (46.2) |  |
| **Age** | | |  | .013 |
| 15–22 | 6 (6.2) | 5 (5.4) | 12 (15.4) |  |
| 23–59 | 62 (63.9) | 73 (79.3) | 49 (62.8) |  |
| >60 | 29 (29.9) | 14 (15.2) | 17 (21.8) |  |
| **Marital status** |  |  |  | <.001 |
| Single | 15 (15.5) | 10 (10.9) | 26 (33.3) |  |
| Married | 82 (84.5) | 82 (89.1) | 52 (66.7) |  |
| **Occupation** |  |  |  | <.001 |
| Indoor | 95 (97.9) | 88 (95.7) | 64 (82.1) |  |
| Outdoor | 2 (2.1) | 4 (4.3) | 14 (17.9) |  |
| **Educational level** |  |  |  | .280 |
| Primary & Junior School | 33 (34.0) | 26 (28.3) | 18 (23.1) |  |
| ≥High School | 64 (66.0) | 66 (71.7) | 60 (76.9) |  |
| **Monthly income (USD)** |  |  |  | .066 |
| <153 | 60 (61.9) | 58 (63.0) | 61 (78.2) |  |
| 153 - 306 | 27 (27.8) | 29 (31.5) | 15 (19.2) |  |
| >306 | 10 (10.3) | 5 (5.4) | 2 (2.6) |  |
| **Religion** |  |  |  | .146 |
| Non-Islam | 1 (1.0) | 3 (3.3) | 5 (6.4) |  |
| Islam | 96 (99.0) | 89 (96.7) | 73 (93.6) |  |
| - 1. **Variables pertaining to the time of the home visit** | | |  |  |
| **Body composition** |  |  |  |  |
| **Metabolic rate (MET)*** |  |  |  | .342 |
| Sleeping/resting/sitting (1.0 MET) | 81 (83.5) | 79 (85.9) | 71 (91.0) |  |
| Standing/light activity (1.5 MET) | 16 (16.5) | 13 (14.1) | 7 (9.0) |  |
| **BMI** |  |  |  | <.001 |
| Underweight | 7 (7.2) | 5 (5.4) | 27 (34.6) |  |
| Normal | 51 (52.6) | 48 (52.2) | 44 (56.4) |  |
| Overweight/obese | 39 (40.2) | 39 (42.4) | 7 (9.0) |  |
| **Clothing insulation (n)** |  |  |  | .749 |
| Trousers, short-sleeve shirt (0.3 clo) | 38 (39.2) | 45 (48.9) | 33 (42.3) |  |
| Trousers, long-sleeve/dress Muslim clothes (0.5 clo) | 58 (59.8) | 46 (50.0) | 44 (56.4) |  |
| Trousers, knit, jacket (0.7 clo) | 1 (1.0) | 1 (1.1) | 1 (1.3) |  |

Outdoor occupation (agriculture/fishery, labour/ manual work). Indoor occupation (student/ retired / homemaker / housewife / civil servant / state enterprise).

Metabolic rate (met): 1 met = 58.2 W/m². (energy produced per unit body surface area by a seated person at rest). Sitting ≈ 1.0 met, standing/light activity ≈ 1.5 met. Clothing insulation (clo): 1 clo = 0.155 m²·K/W. Categories: short‑sleeve ≈ 0.5 clo, long‑sleeve/Muslim dress ≈ 0.7–0.9 clo, jacket ≈ 1.0 clo.

* Chi-squared p-value.

**Table S2.** Housing characteristics by participant group

| **Variable** | **Community (n=97)** | **Non-TB symptomatic (n=92)** | **TB**  **(n=78)** | **P-value^a^** |  |
| --- | --- | --- | --- | --- | --- |
| 1. **Household structural variables** | |  |  |  |  |
| **House type** | |  |  | .189 |  |
| Traditional | 18 (18.6) | 10 (10.9) | 16 (20.5) |  |  |
| Modern | 79 (81.4) | 82 (89.1) | 62 (79.5) |  |  |
| **House attached/detached** | |  |  | .058 |  |
| Attached | 92 (94.8) | 91 (98.9) | 71 (91.0) |  |  |
| Detached | 5 (5.2) | 1 (1.1) | 7 (9.0) |  |  |
| **House stories** |  |  |  | .380 |  |
| 1 storey | 86 (88.7) | 86 (93.5) | 73 (93.6) |  |  |
| 2 storeys | 11 (11.3) | 6 (6.5) | 5 (6.4) |  |  |
| **Floor elevation** | |  |  | .704 |  |
| On ground | 82 (84.5) | 84 (91.3) | 65 (83.3) |  |  |
| 1 meter | 10 (10.3) | 5 (5.4) | 7 (9.0) |  |  |
| 2 meters | 3 (3.1) | 1 (1.1) | 3 (3.8) |  |  |
| 3 meters | 2 (2.1) | 2 (2.2) | 3 (3.8) |  |  |
| **House orientation (front-facing direction)** | |  |  | .856 |  |
| North | 13 (13.4) | 14 (15.2) | 9 (11.5) |  |  |
| Northeast | 10 (10.3) | 9 (9.8) | 6 (7.7) |  |  |
| East | 17 (17.5) | 11 (12.0) | 13 (16.7) |  |  |
| Southeast | 7 (7.2) | 11 (12.0) | 11 (14.1) |  |  |
| South | 15 (15.5) | 13 (14.1) | 13 (16.7) |  |  |
| Southwest | 10 (10.3) | 9 (9.8) | 11 (14.1) |  |  |
| West | 15 (15.5) | 19 (20.7) | 8 (10.3) |  |  |
| Northwest | 10 (10.3) | 6 (6.5) | 7 (9.0) |  |  |
| **Cooking fuel** |  |  |  | .058 |  |
| Clean fuel | 92 (94.8) | 91 (98.9) | 71 (91.0) |  |  |
| Dirty fuel | 5 (5.2) | 1 (1.1) | 7 (9.0) |  |  |
| **Roof material** |  |  |  | .014 |  |
| Tile/ asphalt/+ | 10 (10.3) | 1 (1.1) | 3 (3.8) |  |  |
| Metal | 87 (89.7) | 91 (98.9) | 75 (96.2) |  |  |
| **Ceiling material** | |  |  | .137 |  |
| Wood | 75 (77.3) | 77 (83.7) | 56 (71.8) |  |  |
| Gypsum/Plaster | 11 (11.3) | 3 (3.3) | 11 (14.1) |  |  |
| Other | 11 (11.3) | 12 (13.0) | 11 (14.1) |  |  |
| **Floor material** | |  |  | .326 |  |
| Cement | 30 (30.9) | 34 (37.0) | 28 (35.9) |  |  |
| Wood/other | 9 (9.3) | 2 (2.2) | 5 (6.4) |  |  |
| Ceramic | 58 (59.8) | 56 (60.9) | 45 (57.7) |  |  |
| **Wall material** |  |  |  | .735 |  |
| Cement | 71 (73.2) | 74 (80.4) | 59 (75.6) |  |  |
| Wood | 23 (23.7) | 16 (17.4) | 18 (23.1) |  |  |
| Ceramic/ other | 3 (3.1) | 2 (2.2) | 1 (1.3) |  |  |
| **Living room volume (m^3^)** | | |  | .035 |  |
| ≥9 | 80 (82.5) | 69 (75.0) | 51 (65.4) |  |  |
| <9 | 17 (17.5) | 23 (25.0) | 27 (34.6) |  |  |
| **Living room windows opened** | |  |  | .761 |  |
| Yes | 81 (83.5) | 77 (83.7) | 68 (87.2) |  |  |
| Rarely/ none | 16 (16.5) | 15 (16.3) | 10 (12.8) |  |  |
| 1. **Variables related to home visit** | |  |  |  |  |
| **Month** |  |  |  | <.001 |  |
| September | 11 (11.3) | 4 (4.3) | 19 (24.4) |  |  |
| October | 37 (38.1) | 40 (43.5) | 18 (23.1) |  |  |
| November | 36 (37.1) | 28 (30.4) | 16 (20.5) |  |  |
| December | 13 (13.4) | 20 (21.7) | 25 (32.1) |  |  |
| **Relative humidity (%)** |  |  |  | .394 |  |
| (mean ± SD) | 71.6 ± 5.9 | 70.9 ± 6.5 | 72.2 ± 6.2 |  |  |
| **Air temperature (°C)** |  |  |  | .228 |  |
| (mean ± SD) | 30.9 ± 2.2 | 31.1 ± 2.3 | 30.5 ± 2.4 |  |  |
| **Air speed (m/s)** |  |  |  | .264 |  |
| (mean ± SD) | 0.11 ± 0.36 | 0.06 ± 0.27 | 0.14 ± 0.41 |  |  |
| **CO₂ level (ppm)** |  |  |  | .289 |  |
| (mean ± SD) | 540.5 ± 90.7 | 552.5 ± 90.3 | 562.4 ± 91.0 |  |  |
| **Thermal Sensation Vote (TSV) (subjective thermal comfort)** |  |  |  | .008 |  |
| Slightly cool | 4 (4.1) | 3 (3.3) | 3 (3.8) |  |  |
| Neutral | 8 (8.2) | 11 (12.0) | 21 (26.9) |  |  |
| Slightly warm | 67 (69.1) | 68 (73.9) | 39 (50.0) |  |  |
| Warm | 18 (18.6) | 10 (10.9) | 15 (19.2) |  |  |
| **Predictive Mean Vote (PMV)** | |  |  | .070 |  |
| (mean ± SD) | 1.98 ± 0.93 | 2.11 ± 0.96 | 1.76 ± 0.95 |  |  |

* Chi-squared p-value. Clean fuel (electricity, gas). Dirty fuel (wood, kerosene). Tile/asphalt + (tile, asphalt or another non-metal). Fanger’s Predicted Mean Vote (PMV): Dimensionless index (–3 cold to +3 hot) derived from air temperature, air speed, relative humidity, metabolic rate (met), and clothing insulation (clo). $PMV\boldsymbol{=}\left( 0.303\times e-0.036M+0.028 \right)\times L$

**Table S3.** Mean measured temperature, relative humidity and PMV by thermal perception and health status group.

| **Parameter/health status** | **Comfortable**  **(Mean ± 95% CI)** | **Too warm**  **(Mean ± 95% CI)** |
| --- | --- | --- |
| **Mean temperature** |  |  |
| General community | 28.58 (27.45–29.71) ^b^ | 31.24 (30.82–31.67) ^a^ |
| Symptomatic | 28.44 (27.40–29.49) ^b^ | 31.63 (31.18–32.07) ^a^ |
| TB positive | 28.31 (27.52–29.11) ^b^ | 31.52 (30.99–32.06) ^a^ |
| **Mean relative humidity** |  |  |
| General community | 75.09 (71.91–78.28) ^b^ | 71.15 (69.96–72.35) ^c^ |
| Symptomatic | 79.41 (76.46–82.36) ^a^ | 69.40 (68.15–70.65) ^d^ |
| TB positive | 76.39 (74.14–78.65) ^ab^ | 70.36 (68.86–71.86) ^cd^ |
| **Mean PMV (Predicted Mean Vote)** |  |  |
| General community | 0.96 (0.48–1.44) ^b^ | 2.12 (1.94–2.30) ^a^ |
| Symptomatic | 1.09 (0.64–1.54) ^b^ | 2.30 (2.11–2.49) ^a^ |
| TB positive | 1.07 (0.73–1.41) ^b^ | 2.09 (1.86–2.32) ^a^ |

Values within mean temperature, within mean relative humidity and within mean PMV (each including both thermal sensation types) not having a superscript in common differ significantly at the 5% level (Wald test).
